# Left ventricular perforation by Impella 5.5 during surgery for postinfarction ventricular septal rupture

**DOI:** 10.1186/s43044-024-00579-y

**Published:** 2024-11-06

**Authors:** Hisato Ito, Saki Bessho, Yu Shomura, Keishi Moriwaki, Kaoru dohi, Motoshi Takao

**Affiliations:** 1 Department of Thoracic and Cardiovascular Surgery, Mie University Hospital, 2-174 Edobashi, Tsu, Mie 514-8507 Japan; 2 Department of Cardiology and Nephrology, Mie University Hospital, 2-174 Edobashi, Tsu, Mie 514-8507 Japan

**Keywords:** Postinfarction ventricular septal rupture, Mechanical circulatory support, Impella, Left ventricular perforation, Case report

## Abstract

**Background:**

The perioperative use of the Impella 5.5 has been increasing recently; however, the left ventricular perforation by this device during surgery has not been reported to date.

**Case presentation:**

Postinfarction ventricular septal rupture in a 75-year-old man was successfully repaired with support of a single Impella 5.5 device used for consecutive 28 days perioperatively. The patient underwent surgery after 16 days of Impella support. During surgery, the Impella was left in place expecting its use for left ventricular unloading after the operation. After aortic cross-clamp, when the apex was carefully lifted, the tip of the Impella almost protruded from the posterior wall, and could be seen through the epicardium. The aorta was unclamped briefly, the Impella was pulled out several centimeters, and the aorta was cross-clamped again. The ventricular septal rupture was repaired by the double-layer patch technique via the right ventricle. Immediately before the chest closure, the free wall of the LV ruptured and blood rapidly flowed out. It was where the Impella almost protruded during cardiac arrest, and was repaired with a pledgeted monofilament mattress suture.

**Conclusions:**

A single device can be used throughout perioperative periods; however, if used during surgery, possible risk of left ventricular perforation should be well recognized since the device has no soft pigtail part at its end, and its stiff tip can directly contact the decompressed, flaccid ventricular wall during cardiac arrest.

## Background

Postinfarction ventricular septal rupture (VSR) is infrequent with reported incidence of 0.17–0.31% in the era of primary percutaneous coronary intervention [1]. Despite its rarity, it remains a life-threatening condition. The use of Impella support as a bridge to surgical repair has been reported to effectively decrease mortality in patients with postinfarction VSR [2]. The Impella 5.5 is designed to generate more flow and can be used for longer period compared to its former models. A single device can be used throughout perioperative period; however, if used during surgery, there is a potential risk of left ventricular (LV) free wall rupture. We report a case of LV rupture caused by the Impella 5.5 during surgery, which has not been reported to date.

## Case presentation

A 75-year-old man was diagnosed with postinfarction VSR 10 h after the onset of acute chest pain. Transthoracic echocardiography (TTE) revealed severely hypokinetic anteroseptal wall and an anteroapical VSR 10 mm in diameter with a left to right shunt. Emergency coronary angiography showed totally occluded proximal left anterior descending artery. Right heart catheterization revealed a step up of oxygen saturation from right atrium to pulmonary artery with shunt fraction (Qp/Qs) of 2.7. An Impella 5.5 was placed via a 9-mm Dacron graft attached to the right subclavian artery to stabilize hemodynamics, and to wait for tissue maturation for subsequent surgery. The patient had been awake until he was intubated on 10th admission day when he became delirious. Although he had been supported by the Impella with P6-P8 support level, his heart failure progressed over time. TTE showed that the VSR became slightly larger (13 mm in diameter) than it was on the day of onset, and that moderate tricuspid regurgitation appeared and right ventricular fractional area change decreased from 51 to 42%. There was no shunt reversal on TTE. Atrial fibrillation occurred on 15th admission day, and he underwent surgery the next day.

**Fig. 1:**
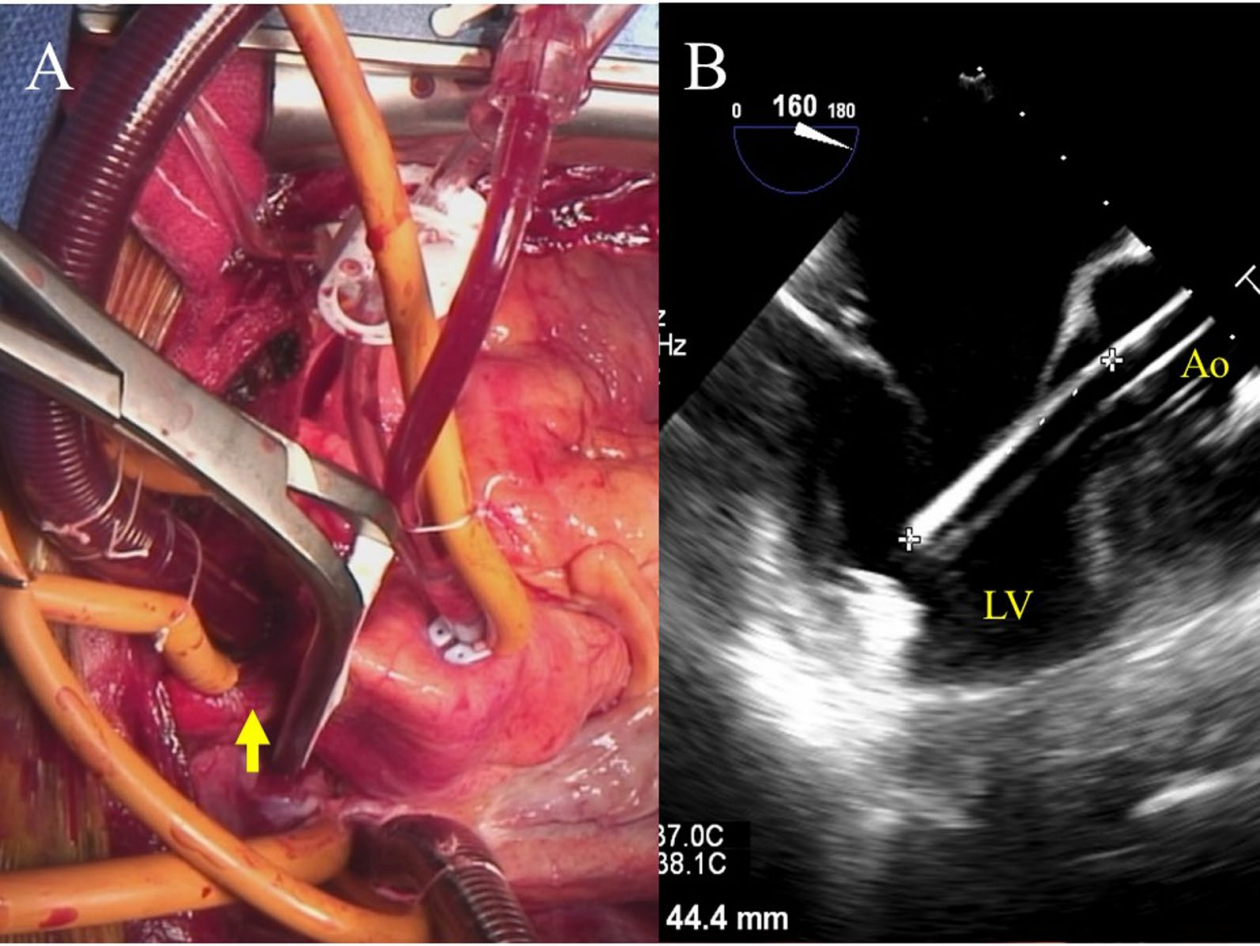
**A** The repeat cross-clamp was applied high enough on the ascending aorta to avoid clamping the motor housing of the Impella 5.5 (arrow, the brachiocephalic artery). **B** The Impella 5.5 was slightly pulled out before the repeat aortic cross-clamp, leaving approximately 5 cm of the tip inside the LV this time. LV, Left ventricle; Ao, Aorta

**Fig. 2:**
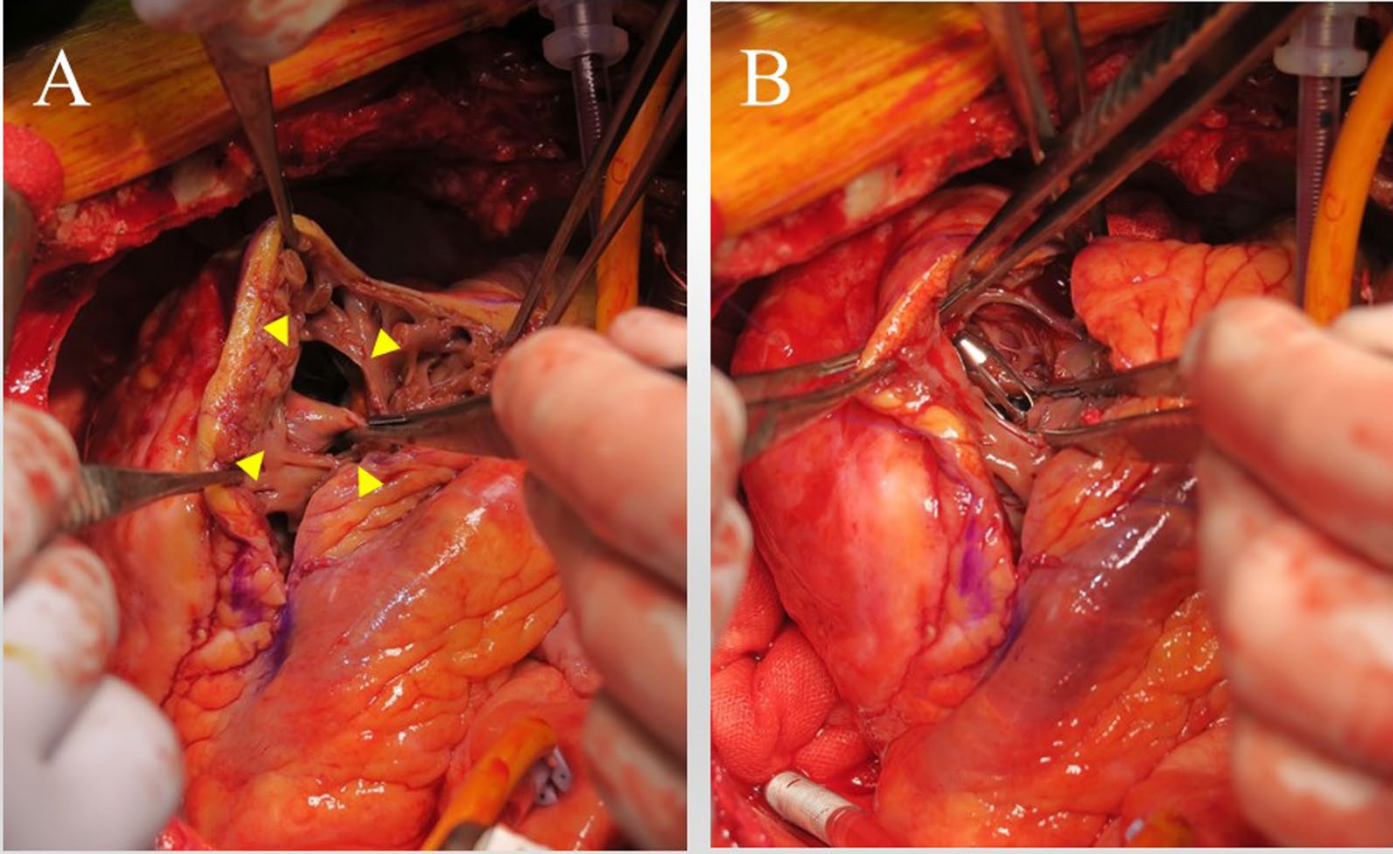
**A** A ventricular septal rupture is noted in the anteroapical ventricular septum (arrow heads). **B** The Impella 5.5 device is lifted up and visible through the defect

**Fig. 3:**
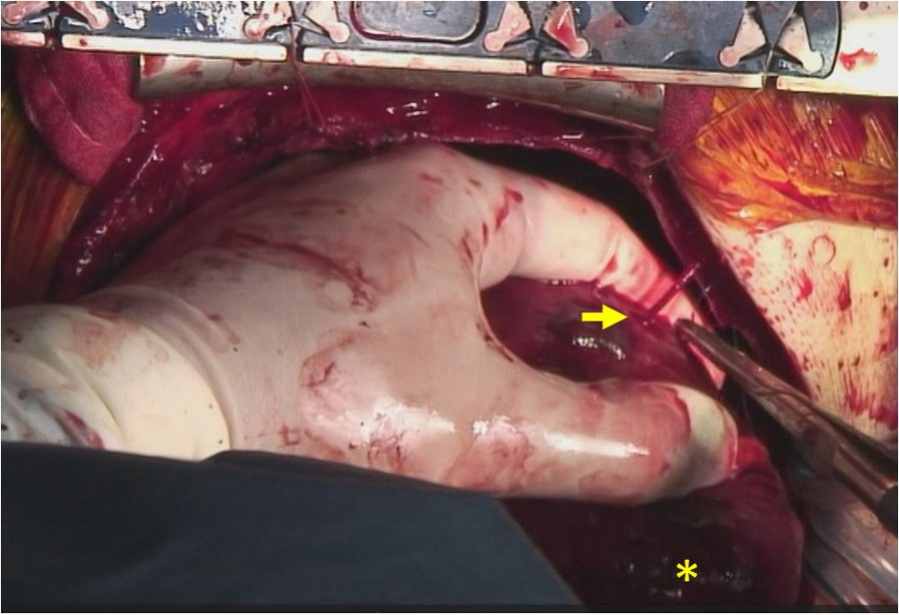
Blood is gushing out from the perforation in the posterior wall of the left ventricle (arrow). The asterisk indicates the apex of the heart

The Impella was left in place expecting its use for LV unloading after the operation. There was a moderate amount of serous pericardial effusion on opening the pericardium. The Impella flow was set to P2 when cardiopulmonary bypass (CPB) was started. The Impella flow was well maintained without an event of suction alarm, which was suggestive of the device contacting the LV wall until aortic cross-clamp. After aortic cross-clamp, the Impella was briefly stopped, and residual blood in the LV was removed by the LV vent. Then, antegrade cardioplegic arrest was obtained while the Impella flow of P2 was maintained. The Impella was set at the ‘surgical mode’ during cardiac arrest. When the apex was carefully lifted, the tip of the Impella almost protruded from the posterior wall, and could be seen through the epicardium. The aorta was unclamped briefly, the Impella was pulled out several centimeters, and the cross-clamp was applied high on the ascending aorta in order to avoid clamping the motor housing of the device (Fig. 1 A, B). The right ventricle was opened, and an approximately 30 × 30 mm VSR was located near the apex (Fig. 2 A, B). The VSR was closed by the double-layer patch technique, and tricuspid valve annuloplasty was performed using the Contour 3D ring (Medtronic, MN, USA). The patient was weaned from CPB with Impella support and nitric oxide inhalation. The CPB time and the aortic cross-clamp time were 177 and 140 min, respectively. The device was not repositioned after aortic unclamping although it had been pulled out a little on cardiac arrest. The Impella flow was set to P6 after weaning off of the CPB, and there was no event of suction alarm throughout the operation. Heparin was fully reversed with protamine, and all the cannulae were removed. Immediately before the chest closure, the free wall of the LV ruptured and blood rapidly flowed out from the posterior wall of the LV. It was where the Impella almost protruded during cardiac arrest, and was repaired with a pledgeted monofilament mattress suture (Fig. 3).

**Fig. 4:**
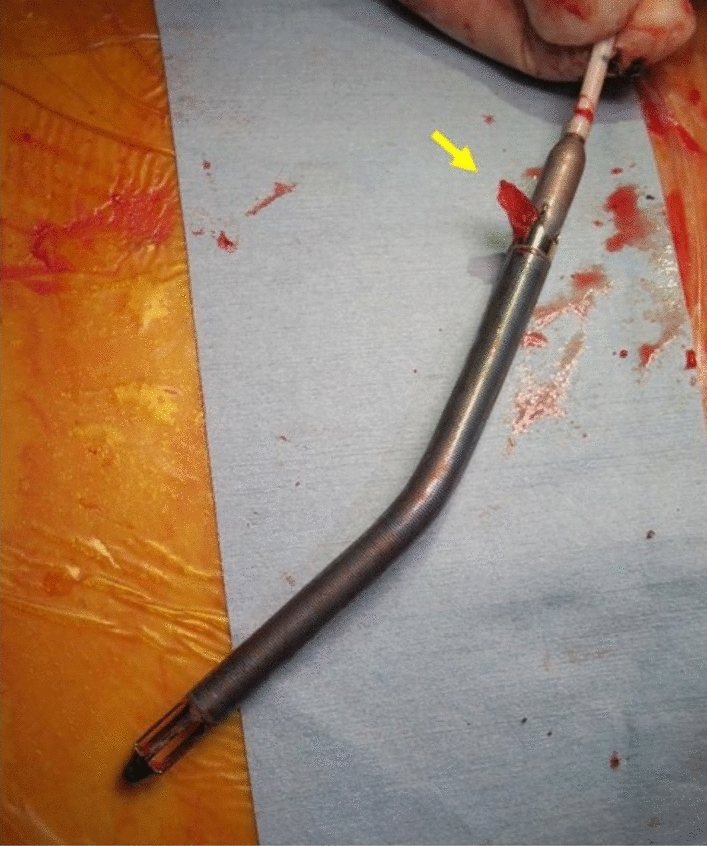
An organized thrombus is attached to the outlet portion of the device (arrow)

Systemic heparinization (10,000 unit/day) was resumed on the postoperative day 1, and then titrated with target activating clotting time of approximately 160 s. The patient was hemodynamically stable with Impella support, nitric oxide, and low dose dobutamine, but one step further away from being weaned from these circulatory supports. Daily TTE showed progressively increasing pericardial effusion. He underwent mediastinal reexploration on postoperative day 12. A large amount of old, dark blood was removed from the pericardial cavity. There was no active bleeding. Once cardiac tamponade was resolved, his hemodynamics improved, and the Impella was removed during surgery. There was an organized thrombus on the blood outlet area (Fig. 4); however, the patient had no thromboembolic events. He was weaned from nitric oxide and extubated on postoperative day 2 and 4, respectively. The patient was discharged home after 88 days of hospitalization. He was doing well in the latest outpatient clinic (16 months after the operation), and TTE showed no residual shunt in the VSR repair site, mild tricuspid regurgitation, and LV ejection fraction of 61%.

## Discussion

Recently, the effectiveness of the Impella device in stabilizing hemodynamics during the perioperative period of postinfarction VSR has been reported [2]. Preoperatively, the Impella device contributes to hemodynamic stabilization, recovery of cardiac muscle from myocardial infarction, and improvement of local myocardial tissue quality. In addition to these effects, its postoperative use is considered beneficial for decreasing the risk of residual leakage of a VSR by LV unloading. The Impella 5.5 is designed to generate more flow and can be used for longer period compared to one of its former models such as Impella CP. We used a single Impella 5.5 device for 28 consecutive days, which is, to our knowledge, the longest period of use for a postinfarction VSR patient among the previously published reports, and is the first report on the LV perforation by the device [3–10].

Preoperatively, whether Impella alone is adequate enough for hemodynamic stabilization depends on the presence or absence of right heart failure. If right heart failure occurs, Impella alone may not work effectively without adequate left ventricular preload. In the present case, right heart failure gradually developed after 10th admission day as the left to right shunt increased due to the VSR becoming larger over time, eventually leading to significant tricuspid regurgitation. In this setting, ECPELLA (extracorporeal membrane oxygenation + Impella) may be effective for hemodynamic support [11]. However, we did not use ECPELLA as it was time for surgery after 16 days of Impella support for myocardial tissue maturation.

During surgery, the shaft of the Impella is clampable along with aortic cross-clamping, allowing for the use of a single device without the need for replacement throughout the perioperative period. In this setting, we usually use epiaortic echocardiography to check the position of a device and not to clamp the motor housing. The Impella 5.5 has a shorter length compared to previous models, which may theoretically reduce the likelihood of the need for aortic cross-clamping too high on the ascending aorta. This device, however, lacks a soft and flexible pigtail part at its end; thus, the stiff inlet part of the device could come into direct contact with the LV wall, which carries a possible risk of free wall rupture as occurred in the present case. One should be cautious especially in cases where an arrested and flaccid heart needs to be lifted. One of the countermeasures to avoid this complication might be to pull out the device so that a minimum of approximately 5 cm of the device is left in the LV, in accordance with the manufacturer’s guidance. Additionally, the aortic cross-clamp should be applied under epiaortic echo guidance not to clamp the motor housing of the device. In the present case, we cross-clamped the aorta as high as possible because epiaortic echo was not available during this repeat aortic cross-clamp. This issue may not be true in beating heart surgeries where the Impella 5.5 tip is not likely to contact the LV wall, or in cases with the previous Impella devices with a pigtail end.

If a single Impella device is used for a prolonged perioperative period, there is a potential risk of thrombus formation as seen in the present case. An organized thrombus was attached to the outlet portion of the Impella device, but fortunately the patient had no thromboembolic events. According to recent review literature, complications associated with long-term use of the Impella device included bleeding, stroke, and device malfunctions, with the duration of support ranged from 9.8 to 70 days. However, the complication observed in the present case was not reported [12]. The importance of balancing bleeding and thromboembolic risks, and maintaining optimal anticoagulation early after surgery is highlighted.

## Conclusions

The Impella 5.5 may have a survival benefit in patients with postinfarction VSR by allowing for prolonged use of a single device throughout the perioperative period. However, the possible risk of LV rupture should be well recognized when the device is continuously used during surgery.

## Data Availability

Data sharing is not applicable to this article as no datasets were generated or analyzed during the current study.

## Abbreviations

VSR: Ventricular septal rupture
LV: Left ventricle
TTE: Transthoracic echocardiography
